# The Association Between Circulating Myostatin and Skeletal Muscle Mass Varies by Nutritional–Inflammatory Status in Hemodialysis Patients

**DOI:** 10.3390/biom16101469

**Published:** 2026-10-09

**Authors:** Xuan Tan, Changsong Ma, Abdullah Md Sheikh, Atsushi Nagai, Shozo Yano

**Affiliations:** 1 Department of Laboratory Medicine, Faculty of Medicine, Shimane University, 89-1 Enya-Cho, Izumo 693-8501, Japan; m259607@med.shimane-u.ac.jp (X.T.); abdullah@med.shimane-u.ac.jp (A.M.S.); 2 Department of Pediatrics, Faculty of Medicine, Shimane University, 89-1 Enya-Cho, Izumo 693-8501, Japan; m259424@med.shimane-u.ac.jp; 3 Department of Internal Medicine 3, Faculty of Medicine, Shimane University, 89-1 Enya-Cho, Izumo 693-8501, Japan; anagai@med.shimane-u.ac.jp; 4 The Center for Integrated Kidney Research and Advance (IKRA), Faculty of Medicine, Shimane University, 89-1 Enya-Cho, Izumo 693-8501, Japan

**Keywords:** myostatin, hemodialysis, chronic kidney disease, sarcopenia, nutritional risk, inflammation

## Abstract

Myostatin (MSTN) is a negative regulator of skeletal muscle growth. However, previous studies in hemodialysis patients have reported inconsistent associations between circulating MSTN and muscle-related parameters, and the reasons for these discrepancies remain unclear. This cross-sectional study aimed to investigate whether these associations varied across nutritional and inflammatory contexts. We enrolled 138 maintenance hemodialysis patients. Serum MSTN levels were measured by ELISA, and skeletal muscle index (SMI), handgrip strength (HGS), and sarcopenia were assessed according to AWGS 2025 criteria. Nutritional and inflammatory status were evaluated using the Nutritional Risk Index for Japanese hemodialysis patients (NRI-JH) and C-reactive protein (CRP), respectively. In the overall cohort, higher serum MSTN levels were associated with greater SMI and HGS. In exploratory analyses, a significant negative three-way interaction among MSTN, NRI-JH, and CRP was observed for SMI (B = −0.445, *p* = 0.018). Above the model-derived Johnson–Neyman boundary of CRP (0.22 mg/dL), the positive association between MSTN and SMI was progressively weaker with increasing NRI-JH scores. These findings suggest statistical heterogeneity in the association between circulating MSTN and skeletal muscle mass across nutritional–inflammatory backgrounds in hemodialysis patients. However, the results are exploratory and require validation in larger independent cohorts.

## 1. Introduction

Sarcopenia is a common and clinically important complication in patients with chronic kidney disease (CKD) undergoing maintenance hemodialysis (MHD). It is characterized by a progressive loss of muscle mass and function and is associated with adverse outcomes including reduced quality of life, falls, hospitalization, cardiovascular events, and all-cause mortality [1,2,3,4]. According to the European Working Group on Sarcopenia in Older People (EWGSOP2), low muscle strength is the entry point for diagnosis and a prerequisite for identifying probable sarcopenia [5]. However, the 2025 update of the Asian Working Group for Sarcopenia (AWGS) explicitly defines sarcopenia as the concurrent presence of low muscle mass and low muscle strength, while physical performance measures are considered outcome indicators rather than diagnostic criteria [6].

Myostatin (MSTN), a member of the transforming growth factor-β (TGF-β) superfamily, is a well-established negative regulator of skeletal muscle growth and differentiation [7]. Experimental studies have demonstrated its role in promoting muscle protein degradation and inducing muscle atrophy [8,9]. Accordingly, MSTN has attracted attention as a potential molecular biomarker and therapeutic target for muscle wasting. However, circulating MSTN concentrations should not be assumed to directly correspond to intramuscular MSTN expression or local signaling activity. In CKD and dialysis populations, circulating MSTN levels may also be influenced by skeletal muscle mass, inflammatory activity, metabolic disturbances, and the uremic and dialysis-related systemic milieu [10]. Indeed, clinical studies have reported inconsistent associations between circulating MSTN and muscle-related outcomes across various populations, including older adults, patients with heart failure, and patients on maintenance dialysis [11,12,13,14,15]. These discrepancies suggest that the relationship between circulating MSTN and skeletal muscle status is more complex. Its relevance to muscle status in patients undergoing MHD therefore remains incompletely understood.

Sarcopenia in CKD develops through multiple interrelated mechanisms, involving uremic toxin accumulation, metabolic acidosis, endocrine disorders, chronic inflammation, and protein-energy wasting (PEW) [16,17,18]. These abnormalities are closely related to the malnutrition–inflammation complex syndrome (MICS), which is associated with muscle wasting and adverse clinical outcomes [19]. Previous studies indicate that inflammatory signaling can activate catabolic pathways in skeletal muscle, whereas nutritional deficiency may impair anabolic and reparative capacity [16,20]. Experimental evidence has also shown that nutritional deprivation can alter MSTN expression in skeletal muscle, while MSTN/activin-related signaling pathways are involved in the regulation of muscle growth and protein metabolism [21,22]. Nutritional risk and inflammatory burden may therefore provide an important biological context in which the association between circulating MSTN and muscle-related parameters varies. However, the direction of such variation is difficult to predict a priori because circulating MSTN may reflect both MSTN-related regulatory processes and the amount of muscle tissue contributing to circulating MSTN levels, as well as other systemic factors. Most previous studies have treated MSTN as an independent correlate of muscle-related outcomes, with limited attention to whether these associations vary across nutritional and inflammatory conditions. Examining these potential interaction effects may help characterize the heterogeneity observed among previous studies and improve the interpretation of circulating MSTN in patients undergoing MHD.

Therefore, the present study aimed to examine the association between circulating MSTN and multiple muscle-related outcomes in patients undergoing MHD, with particular emphasis on the role of nutritional and inflammatory status. Given the uncertainty regarding the direction of these relationships, we explored whether the associations between circulating MSTN and muscle-related outcomes varied across the joint levels of nutritional risk and inflammatory burden.

## 2. Materials and Methods

### 2.1. Subjects

This cross-sectional study was conducted among patients receiving MHD at Himeno Clinic (Shimane, Japan). Patients were eligible if they had been receiving MHD for more than three months. All participants underwent a structured medical history assessment and a standardized physical examination.

Exclusion criteria included (1) acute infection, defined as a physician-diagnosed infection requiring antibiotic treatment or infection-related hospitalization within the preceding 4 weeks, or ongoing antimicrobial therapy at the time of assessment; (2) hospitalization for any other reason or major surgery within the preceding 4 weeks; (3) neuromuscular disease or use of systemic glucocorticoids or exogenous anabolic agents; (4) contraindications to bioelectrical impedance analysis (BIA), such as pacemakers or bilateral limb amputations; (5) decompensated liver cirrhosis or active malignancy. These criteria minimize confounding factors affecting skeletal muscle mass or biomarker interpretation.

During the study period, 168 patients were consecutively assessed for eligibility. Five patients were excluded because they had been receiving hemodialysis for less than three months. Of the remaining 163 eligible patients, 25 were excluded from the present analysis because of incomplete data for one or more variables. Therefore, 138 participants were ultimately included in the final analysis. Ethical approval and written informed consent were obtained as described in the Institutional Review Board Statement and Informed Consent Statement.

### 2.2. Clinical Characteristics and Anthropometric Measurements

Clinical data, including age, sex, dialysis vintage, underlying kidney disease and comorbidities, were obtained from structured medical history assessment and review of electronic medical records. Height and post-dialysis body weight were measured under standardized conditions, and body mass index (BMI) was calculated as weight divided by height squared (kg/m^2^).

### 2.3. Laboratory Measurements

Blood samples were collected before the mid-week hemodialysis session, in the pre-dialysis state, to standardize sampling conditions and minimize potential interference from dialysis-related factors.

Routine laboratory parameters, including blood urea nitrogen (BUN), creatinine (Cr), hemoglobin (Hb), total cholesterol (TC), serum albumin (ALB), and C-reactive protein (CRP), were measured using automated methods at the central laboratory. Dialysis adequacy was assessed using single-pool Kt/V.

Serum samples were stored at −80 °C and subsequently analyzed for myostatin concentration using a commercial enzyme-linked immunosorbent assay (ELISA) kit (Quantikine^®^, R&D Systems, Inc., Minneapolis, MN, USA) according to the manufacturer’s instructions. Briefly, 100 μL of each serum sample was incubated with 50 μL of 1 N HCl for 10 min at room temperature to dissociate the pro-peptide from MSTN. The samples were then neutralized with 50 μL of 1.2 N NaOH/0.5 M HEPES and further diluted with 200 μL of Calibrator Diluent, resulting in a final sample dilution of 1:4. The assay was based on a sandwich ELISA format using monoclonal antibodies specific for mature MSTN. All samples were analyzed in duplicate. According to the manufacturer, the detection range in human serum was 1264–8588 pg/mL and the minimum detectable dose of MSTN ranged from 0.922 to 5.32 pg/mL, mean 2.25 pg/mL. The intra- and inter-assay coefficients were 1.8–5.4% and 3.1–6.0%, respectively. No significant cross-reactivity or interference was observed with Activin RIIA, Activin RIIB, Decorin, GASP-2, GDF-11, or GDF-15, whereas follistatin and GASP-1 may interfere at concentrations > 10 ng/mL. Therefore, the measured concentrations were interpreted as total immunoreactive circulating MSTN rather than as a direct measure of in vivo MSTN biological activity. Inflammatory status was evaluated by serum CRP. Nutritional risk was assessed using the Nutritional Risk Index for Japanese Hemodialysis Patients (NRI-JH) [23], with a higher score indicating greater nutritional risk. Detailed scoring criteria and cutoff values are provided in the Supplementary Methods. NRI-JH scores were conventionally categorized into low-, moderate-, and high-risk according to established cutoff values; these categories were used only to describe the nutritional-risk distribution of the cohort. The geriatric nutritional risk index (GNRI) and creatinine generation rate (CGR) were also calculated using standard formulas [24,25].

### 2.4. Assessment of Body Composition

Body composition parameters, including body fat, fat mass, skeletal muscle mass, and phase angle of the whole body and lower limbs, were assessed using bioelectrical impedance analysis (BIA) (InBody BWA 2.0S, InBody Co., Ltd., Seoul, Republic of Korea) in the post-dialysis state to minimize fluid overload bias. The SMI was calculated as appendicular skeletal muscle mass divided by height squared (kg/m^2^). Muscle strength was measured using a calibrated handheld dynamometer with participants standing and arms fully extended. Two maximal grip tests were performed with each hand, and the highest value was recorded as the final handgrip strength (HGS). For patients with arteriovenous fistula, only the contralateral hand was tested. Low muscle mass and low muscle strength were defined according to the AWGS 2025 consensus criteria [6], with age- and sex-specific cutoff values provided in the Supplementary Methods.

### 2.5. Statistical Analysis

Continuous variables are presented as mean ± standard deviation or median (interquartile range [IQR]), depending on the distribution, and categorical variables are reported as frequencies (percentages). Normality was assessed using the Shapiro–Wilk test along with visual inspection of histograms and Q–Q plots. Between-group comparisons were performed using independent-samples *t*-tests or Mann–Whitney U tests as appropriate. Comparisons among multiple groups were conducted using one-way ANOVA or the Kruskal–Wallis test. Categorical variables were compared using the χ^2^ test or Fisher’s exact test. Comparisons of serum MSTN concentrations across sarcopenia-related groups and comparisons of SMI, HGS, and serum MSTN across nutritional–inflammatory strata were performed using analysis of covariance (ANCOVA), with age and sex included as covariates. Serum MSTN and CRP were log-transformed before analyses because of their skewed distributions. MSTN values were back-transformed and are presented as geometric means.

Age- and sex-adjusted Spearman correlation analyses were performed using a residual-based approach to assess associations between MSTN levels and clinical variables, including subgroup analyses stratified by nutritional and inflammatory status. For exploratory description and visualization of association patterns across different nutritional–inflammatory contexts, participants were stratified into four subgroups based on the medians of NRI-JH score and log-transformed CRP values (Lower NRI-JH/Lower CRP, Lower NRI-JH/Higher CRP, Higher NRI-JH/Lower CRP, Higher NRI-JH/Higher CRP). These data-driven strata were used solely to describe patterns within the present cohort and should not be interpreted as validated clinical risk categories or clinical cutoff values.

Associations between circulating MSTN and muscle-related outcomes were examined using multivariable ordinary least squares regression for the continuous outcomes, SMI and HGS, and multivariable logistic regression for the binary outcome sarcopenia. Moderation analyses were conducted using Hayes’ PROCESS macro for SPSS (version 4.2, Model 3) [26] to examine whether nutritional risk and inflammatory burden jointly moderated these associations. MSTN levels were entered as the independent variable (X), SMI (or HGS/sarcopenia) as the dependent variable (Y), continuous NRI-JH score as the first moderator (W), and CRP as the second moderator (Z). All continuous variables were mean-centered prior to modeling to minimize multicollinearity, and conditional effects were probed at ±1 SD (Supplementary Data S1). When a three-way interaction was statistically significant, the Johnson–Neyman procedure was used to identify the observed range of CRP values over which the conditional MSTN × NRI-JH interaction differed statistically from zero. In addition, the uncertainty of the regression coefficients was assessed using 95% bias-corrected bootstrap confidence intervals based on 5000 resamples.

All statistical analyses were performed using SPSS (version 30.0; IBM Corp., Armonk, NY, USA). Figures were generated using GraphPad Prism (version 8.0; GraphPad Software, San Diego, CA, USA) and Python (version 3.14.0; Python Software Foundation). A two-sided *p* value < 0.05 was considered statistically significant.

## 3. Results

### 3.1. Study Population Characteristics

Of 138 maintenance hemodialysis patients (65.9% men), the median age was 67.5 years (IQR, 60.0–77.0). Baseline characteristics of the study population are summarized in Table 1. The median hemodialysis duration was 67.0 months (IQR, 33.8–177.0). The main etiologies of end-stage kidney disease were chronic glomerulonephritis (33.3%) and diabetic nephropathy (31.9%).

Overall, participants had relatively low muscle mass, with a median skeletal muscle index of 5.9 kg/m^2^ (IQR, 5.3–6.9) and a high prevalence of sarcopenia (52.9%, AWGS 2025). Most patients were classified as low nutritional risk by conventional NRI-JH categories (91.3%), and median CRP was 0.06 mg/dL (IQR, 0.05–0.24). Median serum MSTN concentration was 3.6 ng/mL (IQR, 2.5–5.7). NRI-JH had a significant inverse association with GNRI and CGR.

### 3.2. Serum MSTN Levels According to Sarcopenia-Related Status

**Figure 1:**
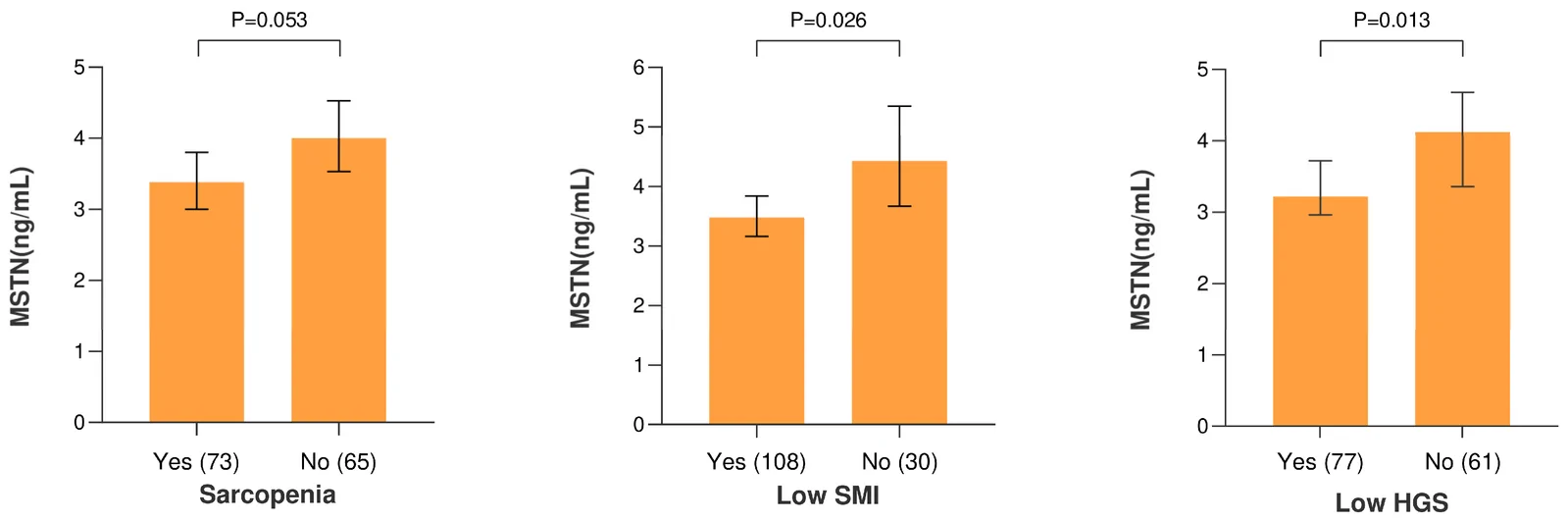
Serum MSTN levels according to sarcopenia-related status. Values are presented as estimated marginal means with 95% confidence intervals derived from ANCOVA models adjusted for age and sex. Statistical significance was defined as *p* < 0.05.

Serum MSTN levels differed according to muscle-related status (Figure 1). Serum MSTN levels were significantly lower in patients with low SMI than in their counterparts with normal SMI [3.48 (3.16–3.84) vs. 4.43 (3.67–5.35) ng/mL, *p* = 0.026]. Similarly, individuals with low HGS exhibited significantly lower serum MSTN levels than the normal HGS group [3.22 (2.96–3.72) vs. 4.12 (3.36–4.68) ng/mL, *p* = 0.013]. In addition, MSTN levels tended to be lower in patients with sarcopenia, but the difference was not statistically significant [3.38 (3.00–3.80) vs. 4.00 (3.53–4.53) ng/mL, *p* = 0.053].

### 3.3. Baseline Characteristics and Differences in the Stratification of Nutritional–Inflammatory Status

Participants were stratified into four subgroups based on the cohort median NRI-JH score and log(CRP) values (Supplementary Table S1). Waist circumference and body fat percentage showed no significant differences across subgroups, whereas biceps circumference exhibited modest but statistically significant variation. Phase angle, a bioimpedance-derived marker of cellular integrity and muscle quality, was lower in the Higher NRI-JH/Higher CRP subgroup. Notably, sarcopenia prevalence also differed significantly across strata and was highest in the two Higher NRI-JH score subgroups.

**Figure 2:**
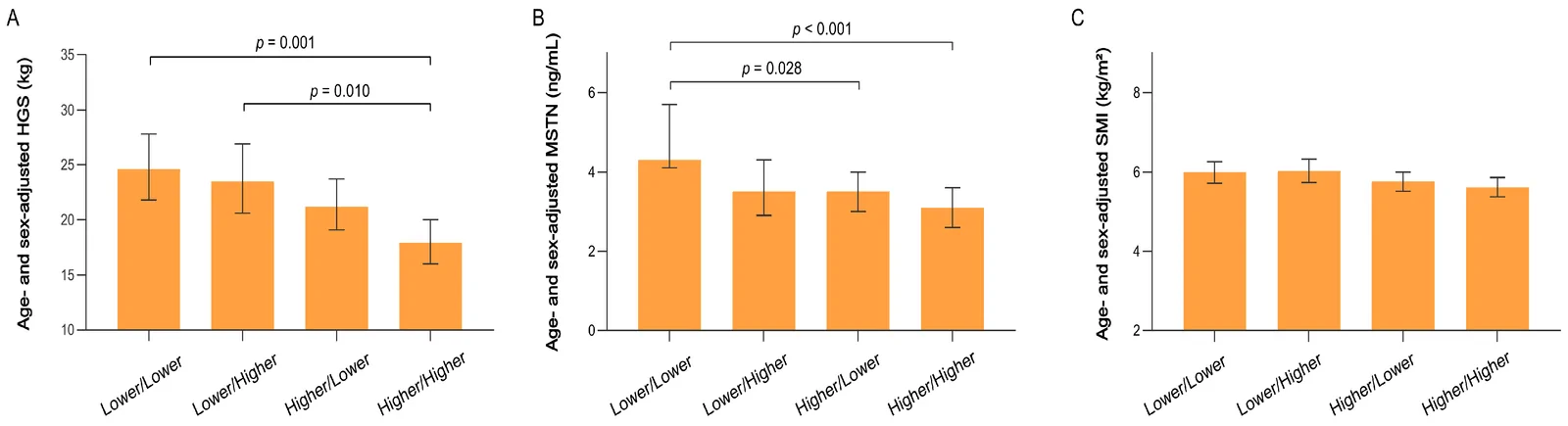
HGS, MSTN, and SMI levels across nutritional–inflammatory status groups. Comparison of (**A**) HGS, (**B**) MSTN, and (**C**) SMI among different nutritional–inflammatory status groups (NRI-JH/CRP). Values are presented as age- and sex-adjusted means with 95% confidence intervals using ANCOVA. *p* values are from Bonferroni-adjusted tests.

After adjustment for age and sex, handgrip strength differed significantly across the nutritional–inflammatory strata and was lowest in the Higher NRI-JH/Higher CRP subgroup (Figure 2A). Serum MSTN levels also differed across strata, with the highest MSTN levels observed in the Lower NRI-JH/Lower CRP subgroup and the lowest levels in the Higher NRI-JH/Higher CRP subgroup (Figure 2B). In contrast, SMI showed no significant differences across the four subgroups (Figure 2C).

### 3.4. Correlations Between Serum MSTN and Clinical/Body Composition Parameters

Age- and sex-adjusted Spearman correlations revealed significant associations between MSTN levels and several clinical and body composition parameters (Supplementary Table S2). Specifically, MSTN levels were positively correlated with SMI (r = 0.297, *p* < 0.001), HGS (r = 0.302, *p* < 0.001), lean body mass (r = 0.246, *p* = 0.004), and phase angle (both whole-body and leg, *p* < 0.001). Conversely, MSTN levels were inversely correlated with CRP (r = −0.309, *p* < 0.001) and NRI-JH score (r = −0.231, *p* = 0.006).

Exploratory stratified analyses revealed differing correlation patterns across nutritional–inflammatory subgroups (Supplementary Table S3). A significant positive correlation between serum MSTN and SMI was observed in the Lower NRI-JH/Higher CRP group (r = 0.54, *p* = 0.004), whereas the correlation with HGS was most prominent in the Higher NRI-JH/Lower CRP group (r = 0.433, *p* = 0.006). These findings suggest that the association between serum MSTN and muscle parameters may vary across nutritional and inflammatory backgrounds.

### 3.5. Associations of Serum MSTN with Muscle-Related Outcomes

Serum MSTN level was inversely associated with sarcopenia in the basic model (OR = 0.131, 95% CI 0.021–0.813, *p* = 0.029). This association was attenuated and no longer significant after additional adjustment for NRI-JH and CRP (OR = 0.248, 95% CI 0.034–1.789, *p* = 0.167), whereas higher NRI-JH score remained associated with greater odds of sarcopenia (OR = 1.244, 95% CI 1.068–1.451, *p* = 0.005) (Table 2).

In analyses of muscle parameters, circulating MSTN remained positively associated with SMI after adjustment for NRI-JH and CRP (B = 1.039, 95% CI 0.471–1.607, *p* < 0.001). Similarly, serum MSTN was positively associated with HGS, and this association also remained significant in the fully adjusted model (B = 6.940, 95% CI 1.368–12.512, *p* = 0.015) (Supplementary Table S4). Given the potential complexity of these relationships and the possible role of nutritional and inflammatory factors, we further conducted moderation analyses.

### 3.6. Moderating Effects of Nutritional and Inflammatory Status

Moderation analyses are summarized in Table 3. A significant negative three-way interaction (MSTN × NRI-JH × CRP) was observed for SMI (B = −0.445, 95% CI −0.812 to −0.078; *p* = 0.018). The corresponding 95% bias-corrected bootstrap confidence interval based on 5000 resamples also excluded zero (95% bias-corrected bootstrap CI: −1.014 to −0.081). The interaction term explained an additional 2% of the variance in SMI (ΔR^2^ = 0.02). In contrast, no significant three-way interaction was detected for HGS (B = −2.174, *p* = 0.223) or sarcopenia (OR = 0.316, *p* = 0.153). None of the two-way interaction terms reached statistical significance (Supplementary Data S1).

**Figure 3:**
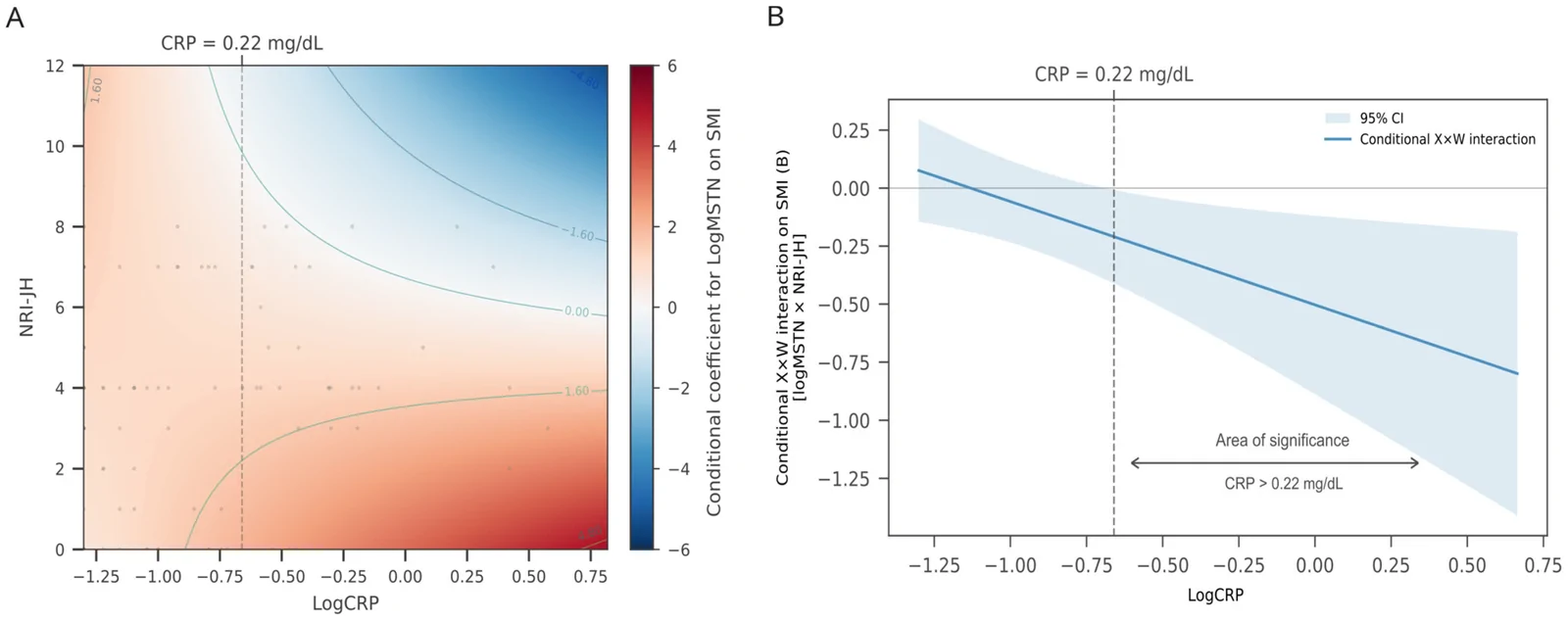
Conditional serum MSTN and SMI association across NRI-JH score and CRP levels. Values displayed in the figure were de-centered for interpretability. (**A**) Heatmap illustrates the estimated conditional regression coefficient for log(MSTN) on SMI across combinations of log(CRP) (x-axis) and NRI-JH (y-axis). Warmer colors indicate a stronger positive association, whereas cooler colors indicate a weaker association. Contour lines represent regions of equal effect. Gray dots represent the distribution of observed samples. Model estimates in sparsely populated regions should be interpreted cautiously. (**B**) The Johnson–Neyman technique identified the region of statistical significance (*p* < 0.05) for the MSTN × NRI-JH interaction across the continuous range of log(CRP). The vertical dashed line marks the model-derived statistical boundary at log(CRP) = −0.66 (corresponding to CRP = 0.22 mg/dL).

As shown in the heatmap (Figure 3A), the conditional association between serum MSTN and SMI varied continuously across nutritional–inflammatory combinations. At lower CRP levels, the association was generally positive across most ranges of NRI-JH score. However, as inflammation increased, the strength of this association varied more markedly according to NRI-JH score. The association remained positive at lower NRI-JH scores but was progressively attenuated and approached the null at the higher end of the observed score range. The Johnson–Neyman procedure identified the range of CRP values over which the conditional MSTN × NRI-JH interaction was statistically significant (Figure 3B). Above the model-derived Johnson–Neyman boundary for CRP (0.22 mg/dL), the positive association between serum MSTN and SMI progressively weakened as NRI-JH score increased, whereas such variation was not statistically evident below this value.

## 4. Discussion

The principal exploratory finding of this cross-sectional observational study was that the association between circulating MSTN and SMI showed statistical heterogeneity across joint levels of nutritional risk and inflammation in patients undergoing MHD. Specifically, the association between MSTN and SMI remained generally positive across most of the NRI-JH range at lower CRP levels, whereas at higher CRP levels this positive association became progressively weaker as NRI-JH scores increased. Nevertheless, the present analysis cannot establish the causal direction of these associations, nor can it demonstrate that nutritional or inflammatory status causally modifies the biological effect of MSTN.

In the overall cohort, higher serum MSTN levels were associated with greater skeletal muscle mass and muscle strength, consistent with previous reports in dialysis populations [27,28]. In the logistic regression analysis, higher serum MSTN was associated with lower odds of sarcopenia only in the basic model, whereas this association was no longer statistically significant after additional adjustment for NRI-JH score and CRP. This suggests that the association between circulating MSTN and sarcopenia may partly reflect broader systemic factors related to nutritional risk and inflammation. Circulating MSTN may partly reflect muscle-derived production or preserved muscle reserve, rather than directly representing local inhibitory MSTN signaling [10,29]. Our previous population-based study also demonstrated an association between circulating MSTN levels and kidney function [30]. Accordingly, particularly in advanced CKD, circulating MSTN levels may reflect multiple interrelated factors. These findings suggest that the interpretation of serum MSTN in future research may need to consider muscle mass and the broader systemic and dialysis-related context [31].

In the present study, serum MSTN levels were inversely associated with both NRI-JH and CRP, and lower concentrations were observed in the exploratory subgroup characterized by higher NRI-JH scores and higher CRP levels. This pattern may be related to reduced skeletal muscle reserve and diminished metabolic capacity under conditions of malnutrition and chronic inflammation [29,31]. It further suggests that the circulating MSTN level may reflect both overall muscle state and the systemic metabolic environment.

These observations are broadly consistent with the concept of the MICS, proposed by Kalantar–Zadeh et al., which emphasizes the synergistic and mutually reinforcing relationship between malnutrition and chronic inflammation [19,32]. This framework provides a biological rationale for considering nutritional risk and inflammatory burden jointly when investigating the association between circulating MSTN and muscle mass.

Against this background, one possible explanation is that circulating MSTN may partly reflect muscle mass or muscle-derived production [10,28,29]. When muscle reserve is relatively preserved, this muscle-derived component may contribute to a clearer positive association between serum MSTN and SMI. In contrast, nutritional impairment and inflammation are associated with reduced muscle reserve, disrupted protein homeostasis, and altered MSTN-related signaling in CKD [9,16,19,20]. Previous experimental studies indicate that pro-inflammatory cytokines may affect MSTN expression through signaling pathways including phosphorylated STAT3 [20,33]. In addition, Smad2/3, Akt/mTOR, and ubiquitin–proteasome-related pathways are all involved in the regulation of muscle protein synthesis and degradation [9,34,35,36]. As nutritional–inflammatory burden increases, circulating MSTN may be increasingly influenced by local or systemic factors other than muscle quantity itself, such that changes in circulating MSTN no longer occur in proportion to changes in actual muscle mass. The correspondence between circulating MSTN and actual muscle mass may be weakened. However, another possibility should also be considered. Catabolic stress, such as poor nutrition and inflammation, most likely induces MSTN secretion, which is rational given nutrient demands [21,22]. In addition, Ibrahim et al. reported higher serum MSTN concentrations in hemodialysis patients with moderate-to-severe malnutrition or muscle wasting [37]. These observations suggest that poorer nutritional status or reduced muscle reserve does not necessarily correspond to lower circulating MSTN. If such adaptive or compensatory regulation becomes prominent, it could theoretically produce a different association between circulating MSTN and muscle-related outcomes. However, because MSTN production and clearance, intramuscular expression, signaling activity, and longitudinal changes in muscle mass were not directly assessed, these interpretations should be regarded as biological hypotheses rather than mechanistic evidence provided by this study.

A significant statistical interaction was observed for SMI but not for HGS or sarcopenia. This may be because MSTN biology is closely related to muscle growth and protein metabolism, which may produce a more direct correspondence with muscle quantity. In contrast, muscle strength is influenced by neuromuscular coordination, physical activity, and comorbidities [38,39], whereas sarcopenia is a composite outcome incorporating both muscle mass and strength [6]. These additional determinants may reduce the detectability of an MSTN-related interaction. Therefore, our results underscore the importance of distinguishing between muscle quantity and muscle function when interpreting circulating MSTN in hemodialysis populations.

From a clinical research perspective, the observed statistical interaction may provide a plausible explanation for the inconsistent associations reported in previous studies. This heterogeneity indicates that the relationship between circulating MSTN and muscle mass warrants evaluation alongside nutritional and inflammatory characteristics in future studies. Whether such an integrated approach improves clinical assessment beyond established measures remains to be determined. Previous studies have shown that comprehensive strategies such as nutritional optimization, inflammation control, and resistance exercise may improve muscle status or related clinical outcomes [17,40,41]. This evidence underscores the importance of considering nutritional and inflammatory background when evaluating muscle health. Although the present findings are insufficient to support treatment selection or individualized risk prediction based on MSTN, they provide a rationale for further evaluation of MSTN within a broader clinical context. Future studies evaluating the potential clinical value of serum MSTN as a muscle-related molecular biomarker should validate these findings in larger longitudinal cohorts, while incorporating a multidimensional framework that considers the nutrition–inflammation–muscle axis.

CRP and NRI-JH were selected as indicators of inflammatory and nutritional status because they are routinely measured and have established relevance to risk assessment in patients undergoing MHD [19,23,42]. CRP is a nonspecific marker of systemic inflammation [43], and previous studies have linked inflammatory activity to muscle-related abnormalities and outcomes in patients with CKD [31,39]. Although these indicators cannot capture the full complexity of the underlying metabolic milieu, they were used to characterize the clinical context in which the association between circulating MSTN and muscle mass was evaluated. The Johnson–Neyman analysis identified a CRP value of 0.22 mg/dL as a model-derived statistical boundary. Although this value falls within the conventional laboratory reference range, it only indicates the point above which the conditional interaction was statistically significant within the present cohort. It should not be interpreted as the onset of a biological effect or as a clinically validated inflammatory threshold. Accordingly, the three-way interaction model, combined with conditional effect visualization, should be viewed as an exploratory framework for characterizing association heterogeneity.

Several important limitations should be acknowledged. First, the cross-sectional design precludes causal inference and limits the ability to determine the temporal sequence among circulating MSTN, nutritional–inflammatory status, and muscle-related outcomes; therefore, reverse causality and residual confounding cannot be excluded. Second, muscle mass was assessed using BIA, which may be influenced by fluid status in hemodialysis patients, although measurements were standardized to the post-dialysis condition to minimize this bias. Third, a single CRP measurement may not fully capture the complexity of the chronic inflammatory milieu. Additionally, although major clinical confounders were adjusted for, some potentially relevant factors, such as physical activity level, dietary intake, and specific pro-inflammatory cytokines (e.g., IL-6, TNF-α), were not included in the analysis. Future studies incorporating these cytokines are warranted to provide complementary mechanistic information. Finally, this was a single-center study with a relatively modest sample size, and observations were sparse in the higher-value regions of the joint NRI-JH–CRP distribution. This uneven data coverage may reduce the precision of the conditional effects estimates in these regions and limit their external generalizability. Therefore, the observed interaction pattern and the model-derived CRP statistical boundary require validation in larger, independent cohorts with broader and more adequately represented distributions of nutritional risk and inflammation.

## 5. Conclusions

In patients undergoing MHD, the association between circulating MSTN and skeletal muscle mass varied by the joint nutritional and inflammatory status. These findings suggest that the interpretation of serum MSTN in future research may need to consider the broader nutritional and inflammatory background. The cross-sectional design limits causal inference. Larger, multicenter longitudinal studies are needed to evaluate the reproducibility and robustness of these findings and to further clarify the clinical relevance of circulating MSTN within the nutrition–inflammation–muscle axis.

## Figures and Tables

**Table 1:** Baseline characteristics of the study population.

Variable	Overall Cohort (*n* = 138)
Age ^†^ (yr)	67.5 [60.0–77.0]
Sex, male, *n* (%)	91 (65.9)
HD duration ^†^ (months)	67.0 [33.8–177.0]
Etiology of ESKD, *n* (%)	
Diabetic nephropathy	44 (31.9)
Chronic glomerulonephritis	46 (33.3)
Hypertensive nephrosclerosis	19 (13.8)
Other causes	29 (21.0)
Diabetes mellitus, *n* (%)	59 (42.8)
BMI ^†^ (kg/m^2^)	20.8 [19.2–22.5]
Lean body mass ^†^ (kg)	37.7 [33.5–43.9]
SMI ^†^ (kg/m^2^)	5.9 [5.3–6.9]
HGS (kg)	24.7 ± 9.1
Waist circumference ^†^ (cm)	80.3 [74.8–88.0]
Biceps circumference ^†^ (cm)	22.7 [20.6–24.4]
Fat mass ^†^ (kg)	14.0 [10.7–17.2]
Body fat (%)	27.0 ± 7.6
Phase angle (whole body)	4.65 ± 0.91
Phase angle (leg)	4.67 ± 1.19
ALB (g/dL)	3.66 ± 0.28
GNRI	93.1 ± 5.8
NRI-JH category, *n* (%)	
Low risk (score 0–7)	126 (91.3)
Medium risk (score 8–10)	9 (6.5)
High risk (score 11–13)	3 (2.2)
PCR (g/kg/day)	0.87 ± 0.16
CGR (mg/min)	104.4 ± 21.1
BUN (mg/dL)	56.2 ± 12.7
Cr ^†^ (mg/dL)	9.32 [8.06–10.75]
Kt/V	1.97 ± 0.33
CRP ^†^ (mg/dL)	0.06 [0.05–0.24]
Hb (g/dL)	11.0 ± 1.09
TC ^†^ (mg/dL)	164.5 [143.3–185.3]
MSTN ^†^ (ng/mL)	3.6 [2.5–5.7]
Sarcopenia, *n* (%)	73 (52.9)

Continuous variables are presented as mean ± SD or median [IQR]; categorical variables are presented as frequencies (%). ^†^ Non-normally distributed variables.

**Table 2:** Multivariable logistic regression analysis of factors associated with sarcopenia.

Variable	Model 1			Model 2		
	OR	95% CI	*p* Value	OR	95% CI	*p* Value
MSTN	0.131	0.021–0.813	0.029	0.248	0.034–1.789	0.167
Sex (male)	2.223	0.872–5.666	0.094	2.279	0.855–6.071	0.099
Age	1.054	1.014–1.097	0.008	1.059	1.017–1.102	0.005
HD duration	1.006	1.002–1.011	0.006	1.007	1.002–1.012	0.006
DM	2.873	1.181–6.987	0.020	3.211	1.277–8.078	0.013
Kt/V	4.092	0.992–16.890	0.051	4.294	0.987–18.692	0.052
NRI-JH (score)	-	-	-	1.244	1.068–1.451	0.005
CRP	-	-	-	0.854	0.343–2.125	0.735

Serum MSTN and CRP were log-transformed before analysis. A *p* value < 0.05 was considered statistically significant. DM, diabetes mellitus.

**Table 3:** Three-way interaction effects of MSTN, NRI-JH, and CRP on muscle-related outcomes.

Outcome	Interaction Term	Effect Size	t/z	95% CI	*p* Value
SMI	MSTN × NRI-JH × CRP	B = −0.445	t = −2.40	−0.812 to −0.078	0.018 ΔR^2^ = 0.02 ^†^
HGS	MSTN × NRI-JH × CRP	B = −2.174	t = −1.23	−5.688 to 1.339	0.223
Sarcopenia	MSTN × NRI-JH × CRP	OR = 0.316	z = −1.43	0.07 to 1.53	0.153

Serum MSTN and CRP were log-transformed before analysis. Models were adjusted for age, sex, diabetes mellitus, HD duration, and Kt/V. ^†^ ΔR^2^ represents the additional variance explained by the interaction term in the linear models. *p* value < 0.05 was considered statistically significant.

## Data Availability

The datasets used and/or analyzed during the current study are available from the corresponding author upon reasonable request.

## Supplementary Materials

The following supporting information can be downloaded at: https://www.mdpi.com/article/10.3390/biom16101469/s1, Supplementary Methods; Table S1. Baseline characteristics of the study population according to nutritional and inflammatory subgroups. Table S2. Age- and sex-adjusted Spearman correlations between MSTN and clinical/body composition parameters. Table S3. Age- and sex-adjusted correlations between MSTN and muscle-related parameters across nutritional–inflammatory subgroups. Table S4. Multivariable linear regression analysis of factors associated with muscle-related parameters. Data S1. Full PROCESS output for the moderation analysis examining the interaction among MSTN, NRI-JH, and CRP in predicting SMI.

## Abbreviations

The following abbreviations are used in this manuscript:

ALB	Serum albumin
AWGS	Asian Working Group for Sarcopenia
BIA	Bioelectrical impedance analysis
BMI	Body mass index
BUN	Blood urea nitrogen
CGR	Creatinine generation rate
CKD	Chronic kidney disease
Cr	Serum creatinine
CRP	C-reactive protein
DM	Diabetes mellitus
ESKD	End-stage kidney disease
GNRI	Geriatric Nutritional Risk Index
Hb	Hemoglobin
HD	Hemodialysis
HGS	Handgrip strength
MICS	Malnutrition–inflammation complex syndrome
MSTN	Myostatin
NRI-JH	Nutritional Risk Index for Japanese hemodialysis patients
PCR	Protein catabolic rate
PEW	Protein-energy wasting
SMI	Skeletal muscle index
TC	Serum total cholesterol
