# Supplementary material for: The Association Between Circulating Myostatin and Skeletal Muscle Mass Varies by Nutritional–Inflammatory Status in Hemodialysis Patients
Source: Biomolecules. 2026 Oct 9;16(10):1469. doi: 10.3390/biom16101469 (PMC13702645; doi:10.3390/biom16101469)
Supplement: Supplementary file 1 [file biomolecules-16-01469-s001.zip › biomolecules-4587835-supplementary.pdf]

Supplementary Materials

Supplementary Methods

Measurements of muscle strength, muscle mass and physical performance according to the AWGS 2025 consensus.

Measurement	Adjustment	Age 50–64 years		Age ≥65 years	
		Male	Female	Male	Female
**Muscle strength**					
Handgrip Strength (kg)	–	<34.0	<20.0	<28.0	<18.0
**Muscle mass**					
DXA	ASM/height^2 (kg/m^2)	<7.2	<5.5	<7.0	<5.4
BIA	ASM/height^2 (kg/m^2)	<7.6	<5.7	<7.0	<5.7
DXA	ASM/BMI	<0.80	<0.55	<0.73	<0.52
BIA	ASM/BMI	<0.90	<0.63	<0.83	<0.57
**Physical performance (outcomes)**					
Five-times chair stand (s)	–	≥10.0	≥10.0	≥12.0	≥12.0
Gait speed, 6-m walk (m/s)	–	<1.2	<1.2	<1.0	<1.0

ASM, appendicular skeletal muscle mass; DXA, dual-energy X-ray absorptiometry; BIA, bioelectrical impedance analysis.

For the patients aged <50 years, the cutoff values for the 50–64-year age group were applied for the present analysis.

Nutritional Risk Index for Japanese hemodialysis patient categories (NRI-JH)

Body mass index	< 20 kg/m^2	Yes = 3	No = 0
Serum albumin level	< 65 years (<3.4 g/dL)<br>≥65 years (<3.2 g/dL)	Yes = 4	No = 0
Serum total cholesterol level	Hypocholesterolemia<br>Hypercholesterolemia	<130 mg/dL=1<br>≥220 mg/dL=2	130–219 mg/dL=0
Serum creatinine level	Female, < 65 years (<9.7 mg/dL)<br>Female, ≥65 years (<8.0 mg/dL)<br>Male, < 65 years (<11.6 mg/dL)<br>Male, ≥65 years (<9.7 mg/dL)	Yes = 4	No = 0

Total Score	Risk Category
0–7	Low
8–10	Medium
11–13	High

Supplementary Table S1: Baseline characteristics of the study population according to nutritional and inflammatory subgroups.

NRI-JH / CRP	Lower/Lower (n=31)	Lower/Higher (n=26)	Higher/Lower (n=39)	Higher/Higher (n=42)	*p* value
**Demographics**					
Age† (yr)	66.0 [57.5–72.0]	70.0 [61.0–78.0]	69.0 [63.0–77.5]	67.5 [59.0–80.0]	0.457
Male, n (%)	16(51.6)	18(69.2)	22(56.4)	35(83.3)	0.017
Diabetes mellitus, n (%)	8(25.8)	11(42.3)	23(59.0)	17(40.5)	0.048
Etiology of ESKD, n (%)					0.931
Diabetic nephropathy	6(19.4)	9(34.6)	15(38.5)	14(33.3)	
Chronic glomerulonephritis	12(38.7)	9(34.6)	11(28.2)	14(33.3)	
Hypertensive nephrosclerosis	5(16.1)	3(11.5)	6(15.4)	5(11.9)	
Other causes	8(25.8)	5(19.2)	7(17.9)	8(21.4)	
**Dialysis-related**					
HD duration† (months)	116.0[73.5-211.5]	71.5 [50.0–184.0]	49.0 [29.0–94.5]	52.0 [29.0–176.0]	0.004
Kt/V	2.11 ± 0.31	1.91 ± 0.33	2.01 ± 0.35	1.87 ± 0.30	0.01
PCR† (g/kg/day)	0.90 [0.83–1.06]	0.91 [0.82–0.99]	0.85 [0.79–0.95]	0.79 [0.65–0.91]	0.001
CGR (mg/min)	121.7 ± 12.3	120.2 ± 14.1	94.8 ± 15.6	90.9 ± 19.2	<0.001
**Anthropometrics / body composition**					
BMI† (kg/m²)	20.7 [19.7–21.9]	21.5 [20.1–23.0]	20.8 [19.0–22.4]	20.4 [18.4–22.7]	0.47
Waist circumference† (cm)	80.8 [74.8–83.9]	80.6 [78.6–91.0]	79.0 [71.3–86.0]	80.7 [76.4–89.4]	0.447
Biceps circumference† (cm)	22.4 [20.5–24.8]	23.6 [22.6–25.2]	22.3 [20.6–23.6]	21.2 [19.9–24.2]	0.033
Body fat (%)	25.6 ± 6.2	28.6 ± 7.5	27.3 ± 7.9	26.6 ± 8.3	0.514
Fat mass† (kg)	13.6 [10.6–15.7]	14.4 [12.7–19.1]	14.1 [10.3–17.6]	13.8 [10.6–19.5]	0.532
Lean body mass (Kg)	39.1 ± 8.6	40.3 ± 7.6	37.5 ± 6.8	39.3 ± 7.8	0.49
SMI (kg/m²)	6.0 [5.7–6.3]	6.0 [5.7–6.3]	5.8 [5.5–6.0]	5.6 [5.4–5.9]	0.090
HGS (kg)	24.6 [21.8–27.8]	23.4 [20.6–26.9]	21.2 [19.1–23.7]	17.9 [16.0–20.0]	<0.001
Phase angle† (Whole Body)	5.36 [4.55–5.75]	4.78 [4.34–5.44]	4.40 [3.94–4.84]	4.08 [3.68–4.62]	<0.001
Phase angle (Leg)	5.43 ± 0.92	5.03 ± 1.07	4.27 ± 1.00	4.26 ± 1.27	<0.001
**Laboratory**					
GNRI	94.3 ± 4.7	93.7 ± 5.4	93.5 ± 5.4	91.4 ± 6.7	0.13
Hb (g/dL)	11.0 ± 1.1	10.8 ± 0.9	11.0 ± 1.1	11.1 ± 1.2	0.842
ALB† (g/dL)	3.7 [3.5–3.9]	3.6 [3.5–3.9]	3.7 [3.5–3.9]	3.6 [3.4–3.7]	0.082
BUN† (mg/dL)	60.2 [54.0–71.1]	57.3 [52.9–65.0]	53.2 [48.5–62.6]	49.4 [39.9–61.1]	0.002
Cr† (mg/dL)	10.76 [9.57–12.30]	10.56 [9.96–12.24]	8.04 [7.17–9.64]	8.55 [7.29–9.34]	<0.001
TC (mg/dL)	176.2 ± 27.9	169.3 ± 40.8	166.7 ± 34.9	152.8 ± 27.2	0.02
MSTN (ng/mL)	4.8 [4.1–5.7]	3.5 [2.9–4.3]	3.5 [3.0–4.0]	3.1 [2.6–3.6]	0.002
**Outcome**					
Sarcopenia, n (%)	9(29.0)	10(38.5)	26(66.7)	28(66.7)	0.001

Continuous variables are presented as mean ± SD or median [IQR]; categorical variables are presented as frequencies (%).

†Non-normally distributed variables. Skewed variables were log-transformed for statistical analysis where applicable. HGS, SMI, and MSTN are presented as estimated marginal means with 95% confidence intervals derived from ANCOVA models adjusted for age and sex. MSTN values were back-transformed and are presented as geometric means.

Supplementary Table S2. Age- and sex-adjusted Spearman correlations between MSTN and clinical/body composition parameters.

Variable	age- and sex-adjusted MSTN	
	r	*p*
BMI (kg/m²)	0.105	0.22
Waist circumference (cm)	0.047	0.589
Biceps circumference (cm)	0.072	0.403
Fat mass (kg)	−0.044	0.611
Body fat (%)	−0.119	0.163
Lean body mass (g)	0.246	0.004
SMI (kg/m²)	0.297	<0.001
ALB (g/dL)	0.12	0.16
PCR (g/kg/day)	0.189	0.026
CGR (mg/min)	0.339	<0.001
BUN (mg/dL)	0.19	0.026
Cr (mg/dL)	0.337	<0.001
Kt/V	0.146	0.088
PA (whole body)	0.507	<0.001
PA (leg)	0.46	<0.001
Hb (g/dL)	0.089	0.299
CRP (mg/dL)	−0.309	<0.001
TC (mg/dL)	0.118	0.167
GNRI	0.168	0.049
HGS (kg)	0.302	<0.001
NRI-JH score	−0.231	0.006

Non-normally distributed variables were analyzed after logarithmic transformation. Spearman correlations were calculated using residuals adjusted for age and sex.

Supplementary Table S3. Age- and sex-adjusted Spearman correlations between MSTN and muscle-related parameters across nutritional–inflammatory subgroups.

NRI-JH / CRP	MSTN–SMI (r)	*p*	MSTN–HGS (r)	*p*
Lower/Lower (31)	0.11	0.556	0.195	0.294
Lower/Higher (26)	0.54	0.004	−0.043	0.833
Higher/Lower (39)	0.248	0.127	0.433	0.006
Higher/Higher (42)	0.125	0.431	0.303	0.051

Spearman correlations were calculated using residuals adjusted for age and sex. Analyses were performed within each nutritional–inflammatory subgroup.

Supplementary Table S4. Multivariable linear regression analysis of factors associated with muscle-related parameters.

Outcome: SMI

Variable	Model 1			Model 2		
	B（95%CI）	*p* value	VIF	B（95%CI）	*p* value	VIF
MSTN	1.280 (0.727 - 1.834)	<0.001	1.157	1.039 (0.471–1.607)	<0.001	1.327
Sex (male)	1.033 (0.752 - 1.314)	<0.001	1.336	1.037 (0.759 –1.315)	<0.001	1.423
Age	−0.027(−0.038 to −0.016)	<0.001	1.210	−0.028 (−0.038 to − 0.017)	<0.001	1.215
HD duration	0.000(−0.002 - 0.001)	0.571	1.507	0.000 (−0.002 to 0.001)	0.601	1.565
DM	0.155(−0.114 - 0.423)	0.257	1.334	0.128 (−0.131– 0.387)	0.329	1.347
Kt/V	−0.771(−1.188 to - 0.353)	<0.001	1.457	−0.770 (−1.172 to − 0.367)	<0.001	1.476
NRI-JH (score)	-	-		−0.073 (−0.113 to − 0.034)	<0.001	1.078
CRP	-	-		0.001 (−0.244 – 0.246)	0.992	1.259

Outcome: HGS

Variable	Model 1			Model 2		
	B（95%CI）	*p* value	VIF	B（95%CI）	*p* value	VIF
MSTN	11.398 (5.804 - 16.992)	<0.001	1.157	6.940 (1.368–12.512)	0.015	1.327
Sex (male)	7.506 (4.669 - 10.343)	<0.001	1.336	8.345 (5.622–11.068)	<0.001	1.423
Age	−0.168 (−0.277 to −0.058)	0.003	1.210	−0.181 (−0.283 to −0.079)	<0.001	1.215
HD duration	−0.016 (−0.028 to −0.003)	0.016	1.507	-0.013 (−0.025 to -0.001)	0.041	1.565
DM	−2.818 (−5.534 to −0.103)	0.042	1.334	-3.349 (−5.887 to -0.811)	0.010	1.347
Kt/V	−4.065 (−8.282 - 0.152)	0.059	1.457	−4.585 (−8.533 to −0.637)	0.023	1.476
NRI-JH (score)	-	-		−0.751 (−1.140 to −0.362)	<0.001	1.078
CRP	-	-		-2.831 (-5.235 to -0.428)	0.021	1.259

Serum MSTN and CRP were log-transformed before analysis. p value < 0.05 was considered statistically significant. DM, diabetes mellitus.

Supplementary Data S1: Full PROCESS output for the moderation analysis examining the interaction among MSTN, NRI-JH, and CRP in predicting SMI.

**************** PROCESS Procedure for SPSS Version 4.2 ****************

Model: 3

Y: SMI

X: Log MSTN

W: NRI_JH

Z: Log CRP

Covariates:

Sex Age DM HD Kt/V

Sample

Size: 138

****

OUTCOME VARIABLE:

SMI

Model

	Coeff	se	t	p	LLCI	ULCI
constant	8.6711	.5722	15.1541	.0000	7.5387	9.8036
Log MSTN	1.1828	.2893	4.0887	.0001	.6102	1.7553
NRI_JH	-.0723	.0213	-3.3985	.0009	-.1143	-.0302
Int_1	-.0876	.0882	-.9930	.3226	-.2621	.0869
Log CRP	.0901	.1355	.6650	.5073	-.1781	.3583
Int_2	.3124	.6323	.4941	.6221	-.9390	1.5637
Int_3	-.0493	.0315	-1.5624	.1207	-.1117	.0131
Int_4	-.4453	.1855	-2.4004	.0179	-.8124	-.0781
Sex	1.0933	.1411	7.7477	.0000	.8140	1.3726
Age	-.0314	.0054	-5.8506	.0000	-.0420	-.0208
DM	.1222	.1286	.9506	.3436	-.1322	.3766
HD	-.0006	.0006	-.9054	.3670	-.0018	.0007
Kt/V	-.6036	.2108	-2.8640	.0049	-1.0207	-.1865

Product terms key:

Int_1: Log MSTN x NRI_JH

Int_2: Log MSTN x Log CRP

Int_3: NRI_JH x Log CRP

Int_4: Log MSTN x NRI_JH x Log CRP

Test(s) of highest order unconditional interaction(s):

	R2-chng	F	df1	df2	p
X*W*Z	.0154	5.7620	1.0000	125.0000	.0179

Conditional effects of the focal predictor at values of the moderator(s):

NRI_JH	Log CRP	Effect	se	t	p	LLCI	ULCI
-3.7899	-.3676	.7795	.5499	1.4176	.1588	-.3088	1.8677
-3.7899	-.2884	.9378	.5075	1.8479	.0670	-.0666	1.9422
-3.7899	.4988	2.5121	.8020	3.1325	.0022	.9249	4.0993
.2101	-.3676	1.0839	.3368	3.2185	.0016	.4174	1.7505
.2101	-.2884	1.1013	.3131	3.5178	.0006	.4817	1.7208
.2101	.4988	1.2735	.4540	2.8053	.0058	.3750	2.1720
3.2101	-.3676	1.3123	.4575	2.8684	.0048	.4068	2.2177
3.2101	-.2884	1.2238	.4282	2.8578	.0050	.3763	2.0714
3.2101	.4988	.3446	.5030	.6850	.4946	-.6509	1.3400

Moderator value(s) defining Johnson-Neyman significance region(s):

Value	% below	% above
.2730	73.9130	26.0870

Conditional X*W interaction at values of the moderator Z:

Log CRP	Effect	se	t	p	LLCI	ULCI
-.3676	.0761	.1064	.7156	.4756	-.1344	.2866
-.2616	.0289	.0966	.2994	.7651	-.1623	.2202
-.1556	-.0182	.0902	-.2023	.8400	-.1968	.1603
-.0497	-.0654	.0878	-.7452	.4575	-.2392	.1083
.0563	-.1126	.0897	-1.2549	.2119	-.2902	.0650
.1622	-.1598	.0958	-1.6687	.0977	-.3493	.0297
.2682	-.2070	.1052	-1.9681	.0513	-.4151	.0012
.2730	-.2091	.1057	-1.9791	.0500	-.4182	.0000
.3742	-.2542	.1171	-2.1697	.0319	-.4860	-.0223
.4801	-.3013	.1310	-2.3006	.0231	-.5606	-.0421
.5861	-.3485	.1462	-2.3844	.0186	-.6378	-.0592
.6921	-.3957	.1623	-2.4379	.0162	-.7169	-.0745
.7980	-.4429	.1792	-2.4720	.0148	-.7975	-.0883
.9040	-.4901	.1965	-2.4936	.0140	-.8790	-.1011
1.0099	-.5372	.2143	-2.5071	.0135	-.9614	-.1131
1.1159	-.5844	.2324	-2.5152	.0132	-1.0443	-.1246
1.2219	-.6316	.2507	-2.5197	.0130	-1.1277	-.1355
1.3278	-.6788	.2692	-2.5218	.0129	-1.2115	-.1461
1.4338	-.7260	.2878	-2.5223	.0129	-1.2956	-.1563
1.5398	-.7731	.3066	-2.5217	.0129	-1.3799	-.1664
1.6457	-.8203	.3255	-2.5204	.0130	-1.4645	-.1762
1.7517	-.8675	.3444	-2.5186	.0130	-1.5492	-.1858

************* BOOTSTRAP RESULTS FOR REGRESSION MODEL PARAMETERS **************

OUTCOME VARIABLE:

SMI

	Coeff	BootMean	BootSE	BootLLCI	BootULCI
constant	8.6711	8.7272	.6140	7.3240	9.8059
Log MSTN	1.1828	1.1886	.3349	.5222	1.8185
NRI_JH	-.0723	-.0703	.0199	-.1135	-.0345
Int_1	-.0876	-.0696	.0869	-.2685	.0671
Log CRP	.0901	.1150	.1548	-.2309	.3599
Int_2	.3124	.3398	.7100	-1.0318	1.7909
Int_3	-.0493	-.0441	.0396	-.1416	.0189
Int_4	-.4453	-.5031	.2612	-1.0139	-.0810
Sex	1.0933	1.0752	.1373	.8337	1.3627
Age	-.0314	-.0320	.0063	-.0432	-.0186
HD	-.0006	-.0007	.0006	-.0017	.0008
DM	.1222	.1151	.1294	-.1274	.3875
Kt/V	-.6036	-.5955	.2293	-1.0623	-.1549

Level of confidence for all confidence intervals in output:

95.0000

Number of bootstrap samples for bias-corrected bootstrap confidence intervals:

5000

W values in conditional tables are the mean and +/- SD from the mean.

Z values in conditional tables are the minimum, the mean, and 1 SD above the mean.
